# Long-term cold storage preservation does not affect fatty livers from rats fed with a methionine and choline deficient diet

**DOI:** 10.1186/s12944-021-01503-y

**Published:** 2021-07-28

**Authors:** Laura Giuseppina Di Pasqua, Clarissa Berardo, Marta Cagna, Barbara Mannucci, Gloria Milanesi, Anna Cleta Croce, Andrea Ferrigno, Mariapia Vairetti

**Affiliations:** 1 Department of Internal Medicine and Therapeutics, University of Pavia, 27100 Pavia, Italy; 2 Centro Grandi Strumenti, University of Pavia, Pavia, Italy; 3 Department of Biology and Biotechnology “Lazzaro Spallanzani”, University of Pavia, Pavia, Italy; 4 Institute of Molecular Genetics, Italian National Research Council (CNR), Pavia, Italy

**Keywords:** fatty acids, fatty livers, marginal livers, methionine and choline deficient diet (MCD), preservation, cold storage, ischemia

## Abstract

**Background:**

Waiting lists that continue to grow and the lack of organs available for transplantation necessitate the use of marginal livers, such as fatty livers. Since steatotic livers are more susceptible to damage from ischemia and reperfusion, it was investigated whether fatty livers with different lipidomic profiles show a different outcome when subjected to long-term cold storage preservation.

**Methods:**

Eight-week-old male Wistar rats fed for 2 weeks by a methionine-choline-deficient (MCD) diet or control diet were employed in this study. Livers were preserved in a University of Wisconsin (UW) solution at 4 °C for 6, 12 or 24 h and, after washout, reperfused for 2 h with a Krebs-Henseleit buffer at 37 °C. Hepatic enzyme release, bile production, O_2_-uptake, and portal venous pressure (PVP) were evaluated. The liver fatty acid profile was evaluated by a gas chromatography-mass spectrometry (GC/MS).

**Results:**

MCD rats showed higher LDH and AST levels with respect to the control group. When comparing MCD livers preserved for 6, 12 or 24 h, no differences in enzyme release were found during both the washout or the reperfusion period. The same trend occurred for O_2_-uptake, PVP, and bile flow. A general decrease in SFA and MUFA, except for oleic acid, and a decrease in PUFA, except for arachidonic, eicosadienoic, and docosahexanaeoic acids, were found in MCD rats when compared with control rats. Moreover, the ratio between SFA and the various types of unsaturated fatty acids (UFA) was significantly lower in MCD rats.

**Conclusions:**

Although prolonged cold ischemia negatively affects the graft outcome, our data suggest that the quality of lipid constituents could influence liver injury during cold storage: the lack of an increased hepatic injury in MCD may be justified by low SFA, which likely reduces the deleterious tendency toward lipid crystallization occurring under cold ischemia.

## Background

Today, liver transplantation remains the only accepted solution for patients suffering from end-stage liver diseases, such as hepatocellular carcinoma, acute fulminant hepatic disorders, hilar cholangiocarcinoma, or metabolic disorders. However, the demand for livers for transplantation vastly exceeds the availability of organs, thus lengthening the already crowded waiting lists around the world [1]. In an attempt to contain this serious situation, many transplant centers have been forced to adopt expanded criteria for the selection of organs to be used, including marginal organs such as: livers from elderly donors, livers from donors after circulatory death (DCD), hepatitis C virus (HCV) positive donors, and steatotic livers [2]. Given the increasing prevalence of nonalcoholic fatty liver disease (NAFLD), steatotic livers represent a valid alternative to meeting the demand for organ transplantation [1]. Hepatic steatosis is categorized as macrovesicular or microvesicular. The latter appears to be less correlated with a poor transplant outcome due to primary non-function (PNF) [3]. Steatosis is also graded as mild (< 33%), moderate (33–66%), or severe (> 66%) [4]. While with a steatosis of 30–60%, a primary non-function (PNF) between 0 and 75% is expected, with steatosis higher than 66%, the success of the outcome becomes difficult to predict. As a consequence, organs are often discarded when the steatosis is associated with other risk factors, such as the advanced age of the donor and prolonged cold storage (CS) preservation time [5].

Fatty livers are more sensitive than healthy livers to CS, especially if it is prolonged (> 12 h) [6]. It is well known that fatty livers are more sensitive to ischemia/reperfusion (I/R) injury [7], thereby causing an increased sensitivity to endotoxin [8], endothelial damage, ATP depletion, necrosis, and sinusoidal swelling and congestion [9, 10]. It has been previously demonstrated in two different models of hepatic steatosis, the methionine and choline-deficient diet (MCD) treated rats and Obese Zucker (*fa/fa*) rats, that the fatty acid profile affects liver outcome after 6 h of CS preservation and 2 h of reperfusion. The analysis of total lipid content and fatty acid constituents revealed that livers from MCD rats were rich in polyunsaturated fatty acids (PUFA) and poor in saturated fatty acids (SFA) when compared with livers from Obese Zucker rats. Thus, considering the deleterious tendency of SFA to produce crystalline structures when exposed to low temperatures, the lipidomic profile of Obese Zucker rats makes them more susceptible to cold ischemia injury when undergoing 6-h CS [11]. Currently, CS is the technique of choice for the preservation of donor livers for transplantation [12]. However, long-term CS preservation can lead to bad outcomes when marginal livers are employed. Since previous data have shown that the fatty acid composition in MCD rats correlated with positive outcomes after 6 h of CS [11], the aim of this study was to investigate the effect of prolonged CS preservation (12 and 24 h) using livers obtained from MCD rats. Moreover, in this work a detailed fatty acid analysis of MCD and control livers was carried out to evaluate its impact on prolonged CS preservation. Given that one of the main limiting factors in using marginal livers after CS is the development of ischemia-related biliary dysfunctions, bile flow was also evaluated. The rate of oxygen uptake, intrahepatic portal venous pressure (PVP), and hepatic enzyme release during the washout and reperfusion were also taken into account. Lastly, the model characterization in terms of lipidomic profiling was also assessed and discussed related to long-term CS preservation.

## Methods

### Animals and experimental design

This work evaluates the outcome of long-term CS preservation and reperfusion in livers from rats fed with a isocaloric control diet in comparison with livers from rats fed with an MCD diet. The use of an animal model was approved by the Italian Ministry of Health and the Pavia University Animal Care Commission (Document number 2/2012). Eight-week-old male Wistar rats (Charles River Laboratories, Italy) were used in this study; they were fed for 2 weeks with a methionine and choline deficient (MCD) diet (Piccioni Laboratory, Italy) or isocaloric control diet (Table 1). The animals were allowed free access to water and food in all the experiments.

**Table 1:** Methionine and Choline deficient diet and Control diet compositions

Ingredients	Formula mg/Kg MCD	Formula mg/Kg Control
Protected Vitamin A 1000	40	40
Vitamin D3 500	5	5
Protected Vitamin E	121	121
L-Alanine	3500	3500
L-Arginine Hydrochloride	12,100	12,100
L-Asparagine Monohydrate	6000	6000
L-Aspartic Acid	3500	3500
L-Cystine	3500	3500
L-Glutamic Acid	40,000	40,000
Glycine	23,300	23,300
L-Histidine Hydrochloride	4500	4500
L-Isoleucine	8200	8200
L-Leucine	11,100	11,100
L-Lysine Hydrochloride	18,000	18,000
L-Phenylalanine	7500	7500
L-Proline	3500	3500
L-Serine	3500	3500
L-Threonine	8200	8200
L-Tryptophan	1800	1800
L-Tyrosine	5000	5000
L-Valine	8200	8200
L-Choline	–	2000
L-Methionine	–	3000
Sucrose	455,414	455,414
Dicalcium phosphate	3000	3000
Vitamin mix	5020	5020
Mineral mix AIN-76	35,000	35,000
Corn Starch	200,000	200,000
Corn Oil	100,000	100,000
Cellulose (Aphacel)	30,000	30,000

Table 1**.** The composition of Methionine and Choline deficient (MCD) diet and Control diet is indicated in mg/Kg diet. The Control diet is identical to MCD diet, but supplemented by L-Methionine and L-Choline.

**Fig. 1:**
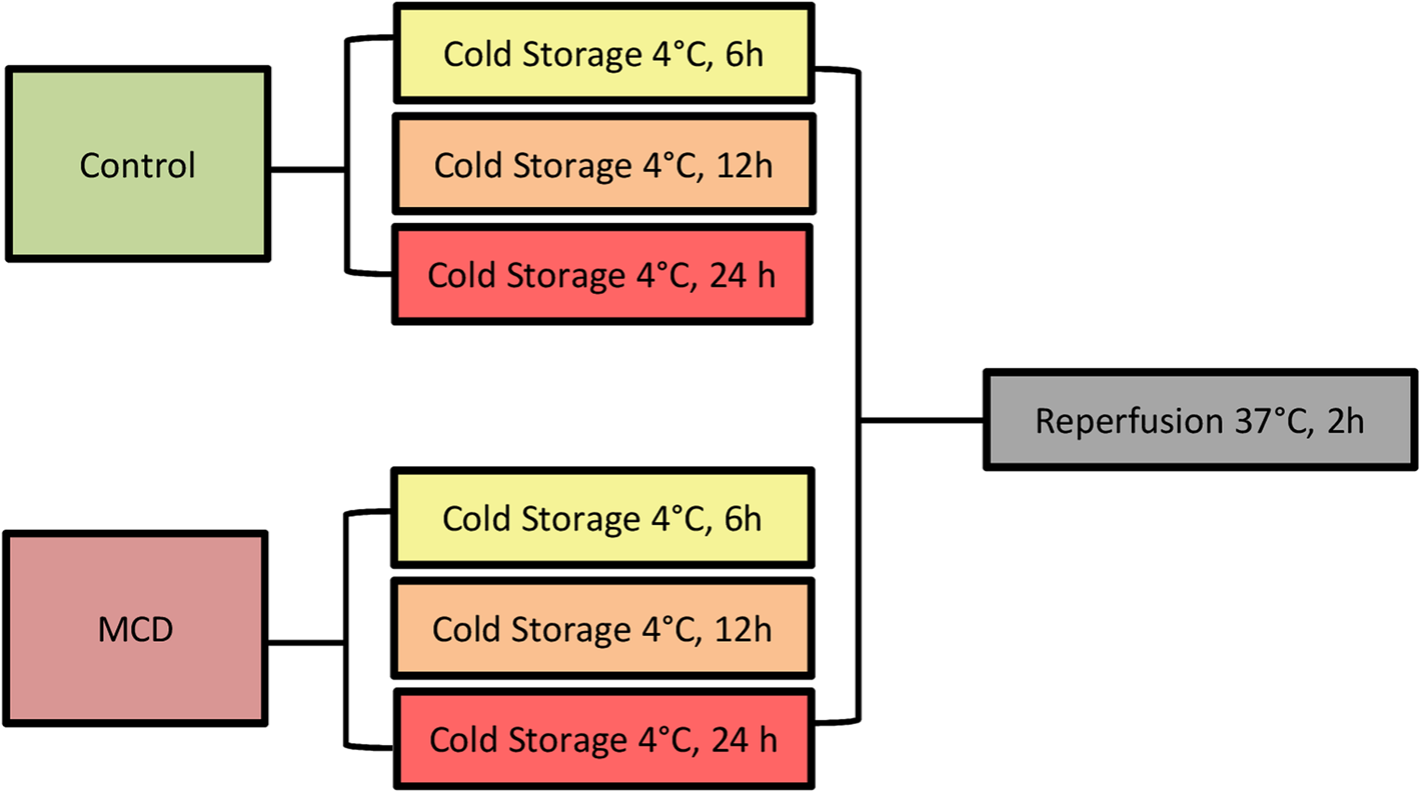
Experimental design. Both livers from control group and MCD group were preserved with University of Wisconsin (UW) solution at 4 °C (Cold Storage, CS) for 6, 12 and 24 h. All organs were reperfused with oxygenated Krebs-Henseleit buffer (O_2_CO_2_, 95 and 5%, respectively) at pH 7.4, for 120 min at 37 °C

After diet administration for 2 weeks, the animals were divided into two groups: the first group was used for fatty acid profile characterization (*n* = 12; 6 MCD, 6 control), the second for long-term CS preservation investigation. The livers from both the MCD and the control rats of the second group were subjected to 6, 12 and 24 h of CS (*n* = 27; 15 MCD, 5/group; 12 control, 4/group) (Fig. 1). The animal abdomen was opened under general anesthesia with sodium pentobarbital (40 mg/kg, intraperitoneally), and liver biopsies and serum samples were collected from the animals in the first group and snap frozen in liquid nitrogen for further analyses. For the second group, the bile duct and the portal vein were cannulated as previously described [13], and the explanted livers were subjected to CS for different periods of time (6, 12, 24 h).

To better adhere to the principle of the three Rs (Replacement, Reduction, Refinement) for the care and use of animals, attention was paid to: i) standardizing the procedures, which were performed by the same operator to avoid operator variability, ii) minimizing animal suffering, iii) minimizing the number of animals, and iv) obtaining the maximum information from each animal.

### Preservation and reperfusion

Static CS preservation was performed after liver isolation and washing with 50 mL of Ringer Lactate. The organs were flushed with Belzer-University of Wisconsin solution at 4 °C (UW, ViaSpanTM, DuPont Pharmaceuticals, Wilmington, DE, USA) for 2 min and then preserved in this solution, considered the gold standard for liver preservation, for 6, 12 or 24 h at 4 °C. Following CS, the livers were kept at room temperature for 10 min and then reperfused with oxygenated (O_2_CO_2_, 95 and 5%, respectively) Krebs-Henseleit buffer at pH 7.4 for 120 min at 37 °C [11]. The reperfusion flux was kept constant throughout reperfusion and the oxygen dissolved in the influent perfusate was measured using a Clark-type oxygen electrode, while the portal venous pressure was constantly monitored with a water column connected to the portal vein inflow cannula. The starting perfusion pressure was about 11–12 mmHg. The bile was monitored and stored in polystirene dark tubes during the reperfusion time.

### Biochemical parameters

The determination of the aspartate aminotransaminase (AST, mU/mL) and alanine aminotransferase (ALT, mU/mL) release in serum, washout, and perfusate was assessed using ELISA kits purchased from Abcam (Cambridge, UK) and following the manufacturer’s instructions. The lactate dehydrogenase (LDH, mU/min/g) release in the washout and perfusate was assessed spectrophotometrically at a wavelength of 340 nm, measuring its activity by the addition of a saturating concentration of NADH and pyruvate, as previously described [14].

### Lipid peroxidation

The extent of lipid peroxidation was evaluated using the Esterbauer and Cheeseman [15] method, in terms of thiobarbituric acid reactive substance (TBARS) formation, as previously described [16]. TBARS concentrations were calculated using malondialdehyde (MDA) as standard.

### Liver morphology

Before preservation and at the end of reperfusion, liver biopsies were rapidly collected, dried on a paper towel, and fixed in 2% p-formaldehyde in 0.1 M PBS, pH 7.4 for 24 h. The biopsies were then processed routinely until they were embedded in Paraplast wax. Paraplast-embedded liver sections were cut at 6 μm and stained with Hematoxylin and Eosin (H&E) for histological examination [17]. Hepatic steatosis was characterized quantitatively (percentage of hepatocytes containing lipid droplets) and qualitatively, according to the size of the droplets (micro- and macrosteatosis) [18].

### Hepatic collagen deposition

The collagen deposition evaluation was performed by detecting collagen fibers by means of Sirius red (Direct Red 80) staining [19] before preservation. Unfixed tissue sections were air-dried for 48 h and then immersed for 1 h in the staining solution (0.1% of Sirius red in picric acid-saturated aqueous medium) and washed in two changes of acidified water (0.5% acetic acid).

### Hepatic lipid extraction and total lipid content determination

Hepatic lipid extraction was assessed according to the method proposed by Lyn-Cook et al. [20]. Frozen liver biopsies (70–100 mg each) were homogenized in water (200 μL), and lipids were extracted by incubating each sample with 1 mL chloroform-methanol (2:1), at room temperature with intermittent agitation for 1 h. After centrifugation at 3000 rpm for 5 min, the lipid-containing lower fraction was obtained, transferred to a clean tube, and dried under constant flow of N_2_. Pellets were re-suspended in 100 μL of 100% ethanol. Aliquots of lipid extract were transferred to a 96-well polystyrene black plate, with the addition of phosphate buffered saline (PBS) 1X and Nile Red solution (1 mg/mL in DMSO) to each well, to quantify total lipid content. A Victor^2^ Multilabel Counter microplate reader (Perkin Elmer, Milano, Italy) was used for fluorescent intensity measurement (excitation/emission: 485/572 nm). The measurement results were presented as total lipid/liver weight (mg/g).

### Fatty acid characterization

The fatty acid profile of liver extracts was analysed by means of a ThermoFisher Scientific DSQII GC/MS system (TraceDSQII mass spectrometer, TraceGCUltra gaschromatograph), Xcalibur MS Software Version 2.1 (including NIST Mass Spectral Library, NIST 08), and Wiley Registry of Mass Spectral Data 8th Edition for the assignment of chemical structures to chromatographic peaks, as previously described [21]. Each identified peak was expressed as relative percentage areas of total methylated fatty acids (FAME). The reference standard was the Marine Oil FAME Mix from Restek (Superchrom S.r.l., Cernusco sul Naviglio, Milan, Italy). Dichloromethane was used as a blank to avoid carryover from the previous analysis.

### Statistical analysis

The statistical analysis was done using MedCalc Statistical Software version 18.11.3 (MedCalc Software bvba, Ostend, Belgium; https://www.medcalc.org; 2019). Statistical analysis was performed with a T-Test, when appropriate, or a one-way ANOVA with Tukey’s multiple comparison test, as a post-hoc test, or the Kruskall-Wallis and Dunn’s test, as appropriate. The analysis of multiple measures was performed using a non-linear mixed model. To assess the normality of variance changes, the Kolmogorov-Shapiro normality test was used. The results are expressed as a mean value ± standard error (SE). The value of *P ≤ 0.05 was considered the criterion for statistical significance.

## Results

### Cold storage preservation and reperfusion injury in livers from MCD rats

**Fig. 2:**
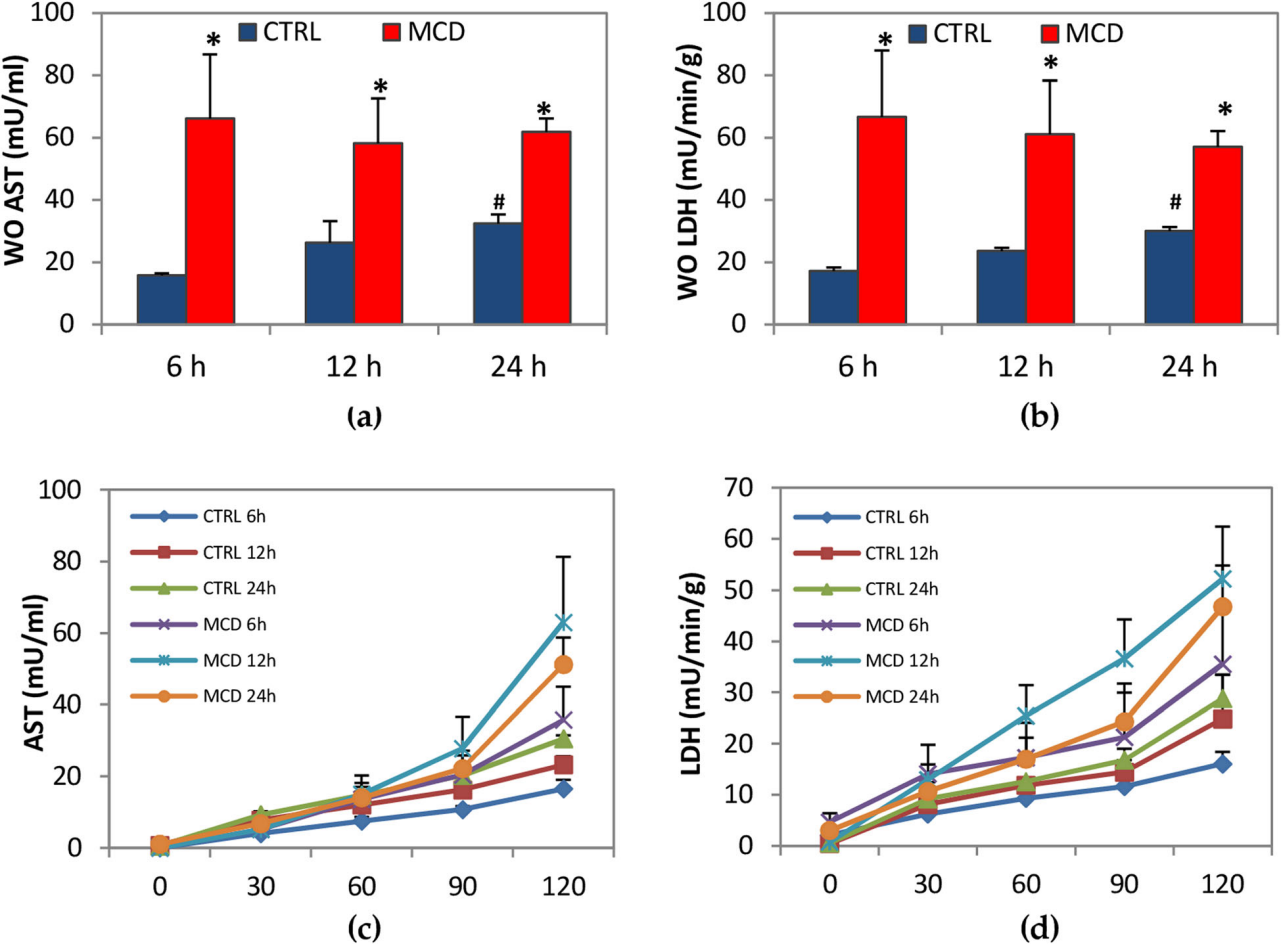
Enzyme release after the washout (WO) and during the reperfusion. Livers were preserved for 6, 12 or 24 h by CS, followed by 120 min reperfusion. (**a**) aspartate aminotransaminase (AST) in the WO; (**b**) lactate dehydrogenase (LDH) in the WO; (**c**) AST during reperfusion; (**d**) LDH during reperfusion. The data are presented as mean ± SE, *n* = 5/MCD groups, *n* = 4/control groups. For figure (**a**) and (**b**) the *P* value was calculated by one-way ANOVA with Tukey’s multiple comparison test. For figure (**c**) and (**d**) the *P* value was calculated by non-linear mixed models. *P ≤ 0.05 MCD vs its respective control; #*P* ≤ 0.05 control 24 h vs control 6 h

When comparing livers obtained from MCD rats, no differences in enzyme release were found after 6-, 12- or 24-h CS preservation both during the washout or reperfusion (Fig. 2). Higher levels in AST and LDH were detected in MCD rats when compared with their respective control groups after washout. A significant increase in AST and LDH release was found in control livers preserved for 24 h when compared with those preserved for 6 h (Fig. 2A and B). During 120 min of reperfusion, no significant differences in enzyme release were detected comparing livers from MCD rats with the respective control group (Fig. 2C and D).

**Fig. 3:**
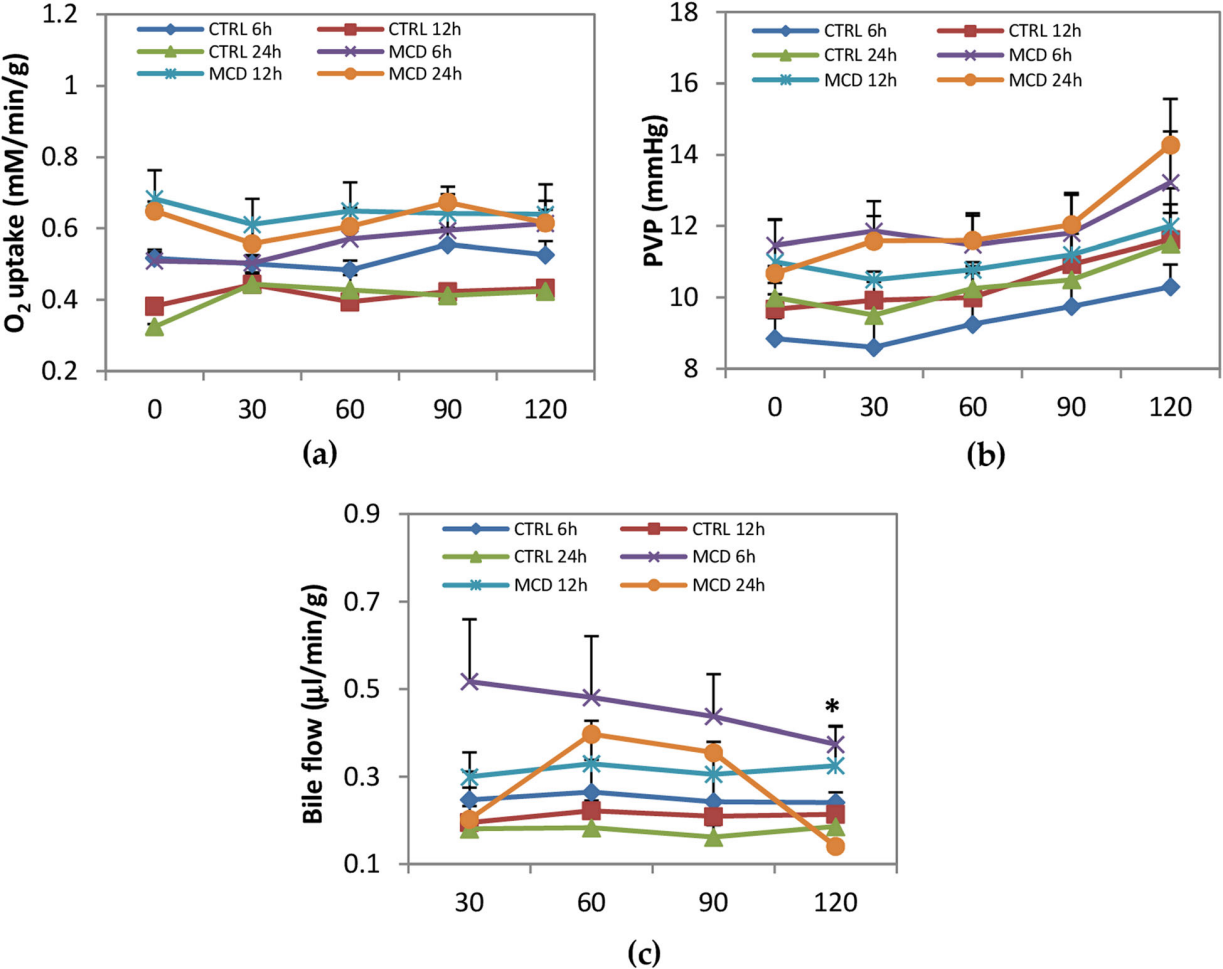
Oxygen uptake, intra-hepatic portal venous pressure (PVP) and bile flow production during reperfusion. Livers were preserved for 6, 12 or 24 h by CS, followed by 120 min reperfusion. (**a**) Oxygen uptake; (**b**) PVP; (c) bile flow. The data are presented as mean ± SE, n = 5/MCD group, n = 4/control group. The *P* value was calculated by non-linear mixed models. *P ≤ 0.05 MCD 6 h vs control 6 h at 120 min reperfusion

Oxygen uptake, intra-hepatic portal venous pressure, and bile secretion were evaluated as parameters describing liver functionality (Fig. 3). Comparing livers obtained from MCD rats preserved for 6, 12, or 24 h of CS, no significant differences were observed in oxygen uptake. No differences were detected also when MCD rats were compared with their respective controls: the oxygen uptake of both the MCD and the control livers during 120 min of reperfusion was between 0.45 mM/min/g and 0.7 mM/min/g (Fig. 3). During the reperfusion, a similar trend was found in PVP, with no significant difference among MCD livers cold-preserved for 6, 12, or 24 h and between MCD and their respective controls. No changes were observed in the bile flow during reperfusion when comparing MCD livers preserved for 6, 12, or 24 h. A significant increase in bile production was only found after 120 min of reperfusion in MCD livers preserved for 6 h when compared with their respective controls (Fig. 3C).

**Fig. 4:**
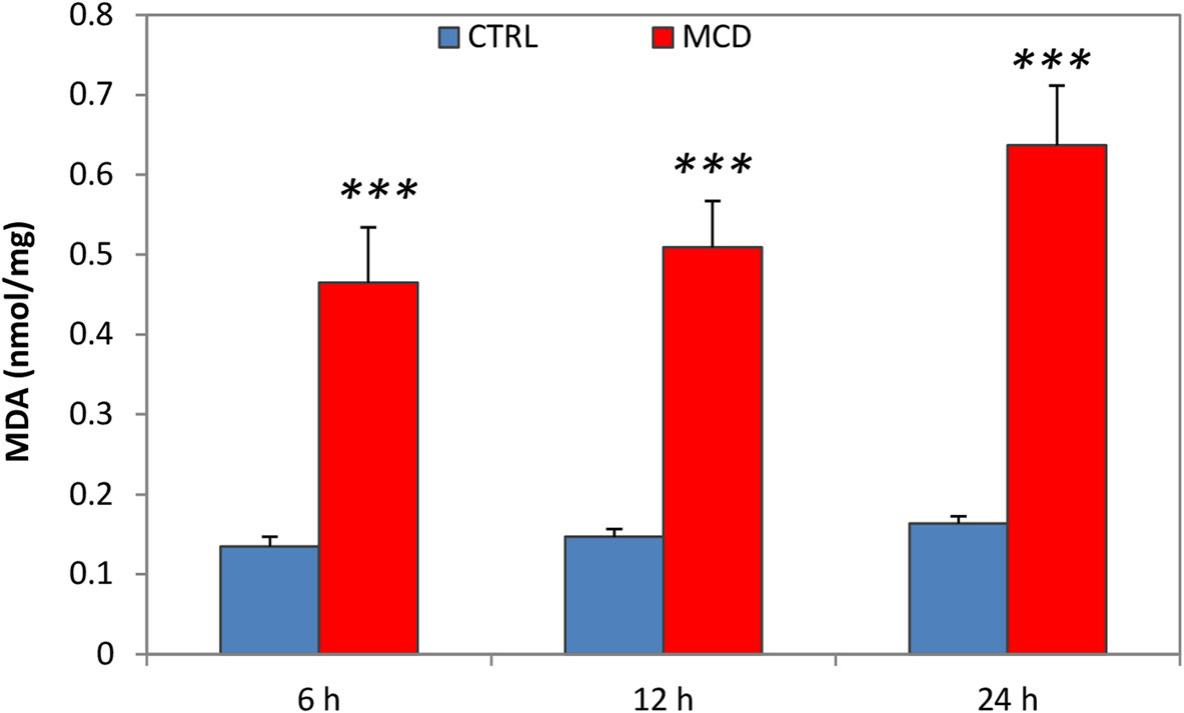
Lipid peroxidation after reperfusion. Livers of both MCD and control rats were preserved for 6, 12 and 24 h by CS, followed by 120 min reperfusion. Thiobarbituric acid reactive substance (TBARS). The data are presented as mean ± SE, n = 5/MCD group, n = 4/control group. The *P* value was calculated by one-way ANOVA with Tukey’s multiple comparison test. ***P ≤ 0.001 MCD vs its respective control

Oxidative stress induced by ischemia and reperfusion injury was evaluated in terms of TBARS formation. In particular, the production of MDA was measured (Fig. 4). Comparing livers obtained from MCD rats and preserved for 6, 12, or 24 h of CS, no significant differences were detected in MDA formation among the three groups. A significant difference was observed when MCD livers were compared with their respective controls, at all considered times (Fig. 4).

### Liver morphology

**Fig. 5:**
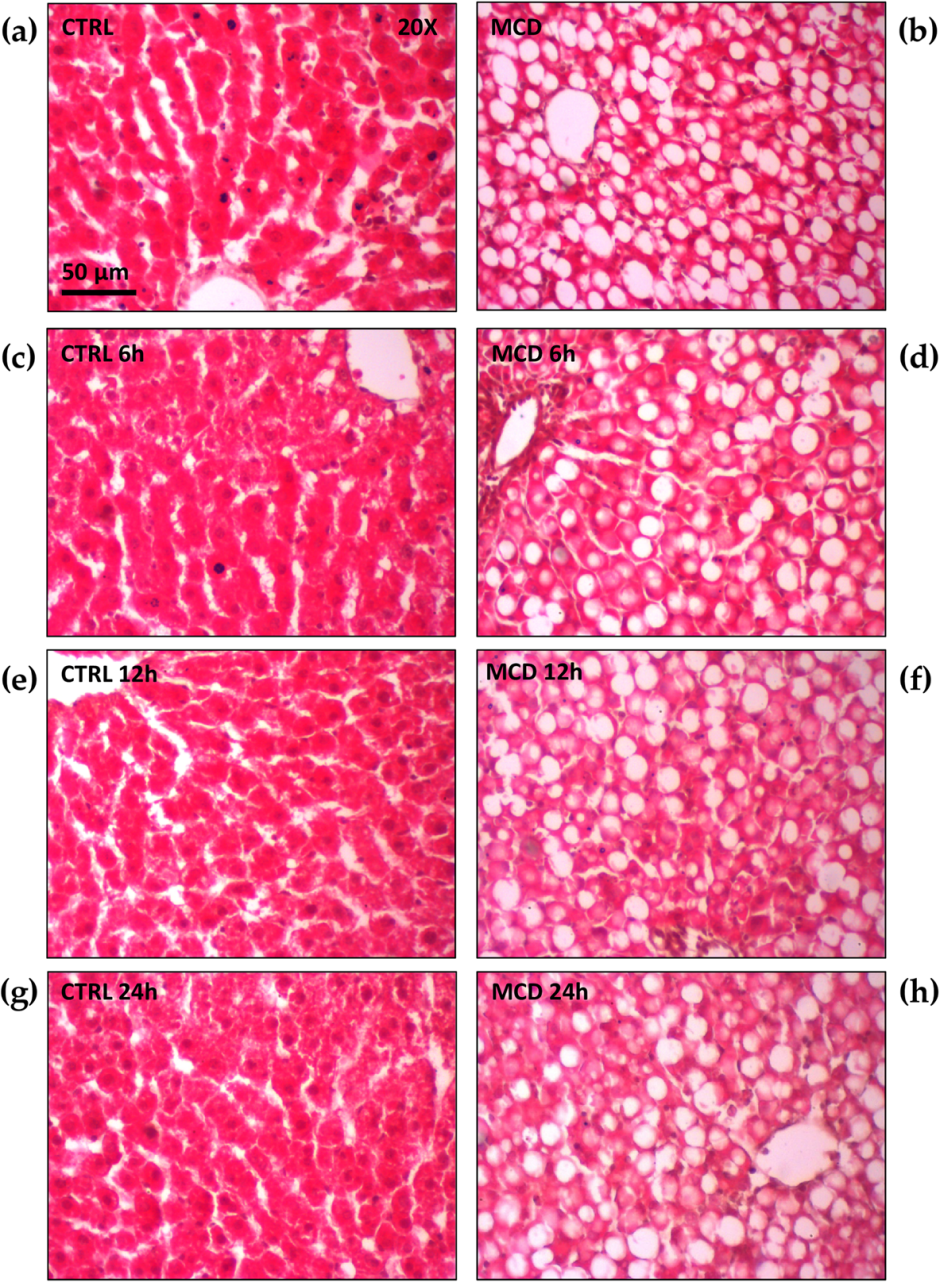
Liver morphology obtained by H&E staining after 6, 12 or 24 h of CS preservation both in control and in MCD fed rats. (**a**) Control liver after 6 h of CS; (**b**) MCD liver after 6 h of CS; (**c**) control liver after 12 h of CS; (**d**) MCD liver after 12 h of CS; (**e**) control liver after 24 h of CS; (**f**) MCD liver after 24 h of CS. Scale bar: 50 μm

A morphological evaluation of the samples obtained from both the MCD and control rats was performed before preservation and after 6, 12, or 24 h of CS, respectively. As expected, the liver morphology obtained by H&E staining revealed a strong difference between livers from control rats and MCD rats. Control rats showed well-preserved hepatic architecture for all CS time periods, while the liver morphology of MCD livers displayed lipid droplets within the hepatocytes. No significant differences were detected in the morphology of livers from MCD rats before preservation and after CS for 6, 12, or 24 h (Fig. 5). Hepatic steatosis was characterized quantitatively as the percentage of hepatocytes containing lipid droplets (Controls < 5%, MCD: 48.7% ± 1.9) and qualitatively according to the size of the droplets distinguishing macro- and microsteatosis (macrosteatosis: 72.2%, microsteatosis: 28.8%).

### Hepatic collagen deposition

**Fig. 6:**
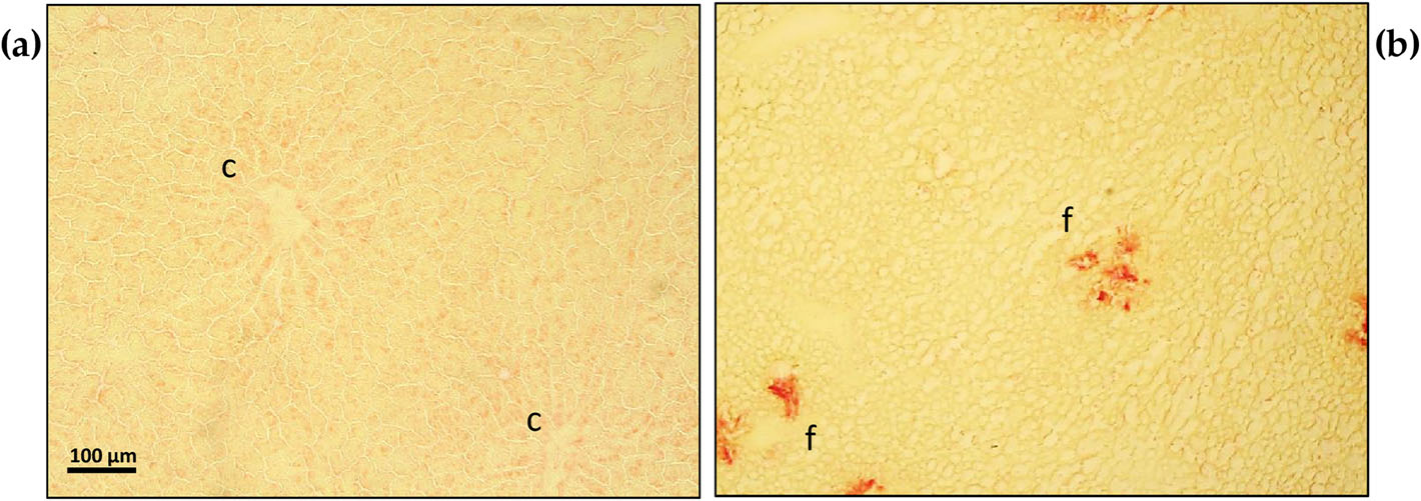
Hepatic collagen deposition evaluation obtained by Sirius red staining before preservation both in control and in MCD fed rats. (**a**) control liver: yellowish hepatocytes can be appreciated organized in anastomozing cords, converging towards the central veins (**c**); (**b**) MCD liver results in the presence of lipid droplets and initial foci of collagen deposition, positive to red staining by Sirius red (**f**). Scale bar 100 μm

The analysis of tissue sections stained by Sirius red showed the presence of an initial collagen deposition appearing as the foci of red fibrous structures within the yellowish parenchyma of livers from the MCD rats when compared with the control livers (Fig. 6).

### Liver fatty acid profile

An accurate characterization of the fatty acid profile was performed in MCD rats in comparison with the control group. The gas chromatography-mass spectrometry (GC/MS) analysis focused on the presence of 21 specific fatty acids in our rat model. Differences in the percentage of saturated fatty acids (SFA), monounsaturated fatty acids (MUFA), and polyunsaturated fatty acids (PUFA) were found when comparing MCD rats with rats fed by an isocaloric control diet (Table 1). As regards SFA, a significant decrease in MCD rats in the myristic (C14:0), stearic (C18:0), arachidic (C20:0), behenic (C22:0), and lignoceric (C24:0) acids was detected when compared with the control rats; no changes in the pentadecanoic (C15:0) and palmitic (C16:0) acids were found when comparing the MCD and the control rats. In the MUFA group, higher levels of oleic (C18:1n), veccenic (C18:1), and nervonic (C24:1) acids were observed in MCD compared to the control rats; among PUFA, linoleic (C18:2), linolenic (C18:3), eicosatrienoic (C20:3), docosatetraenoic (C22:4), and docosapentaenoic (C22:5) acids were significantly augmented in the MCD group with respect to the control group. On the contrary, a decrease in eicosadienoic (C20:2), arachidonic (C20:4), and docosahexaenoic (C22:6) acids was found in MCD rats compared with the control rats (Table 2).

**Table 2:** Fatty acid characterization in MCD rats versus control rats

SFA		Control (%)	MCD (%)	*P
C14:0	Methyl Myristate	0.86 ± 0.12	0.57 ± 0.05	*
C15:0	Methyl Pentadecanoate	0.13 ± 0.03	0.20 ± 0.03	ns
C16:0	Methyl Palmitate	26.20 ± 1.38	23.56 ± 0.68	ns
C18:0	Methyl Stearate	14.82 ± 1.53	6.46 ± 0.42	***
C20:0	Methyl Arachidate	0.24 ± 0.09	0.06 ± 0.01	*
C22:0	Methyl Behenate	0.15 ± 0.01	0.04 ± 0.004	***
C24:0	Methyl Lignocerate	0.18 ± 0.03	0.06 ± 0.02	*
**MUFA**				
C16:1	Methyl Palmitoleate	1.54 ± 0.17	1.10 ± 0.18	ns
C18:1n	Methyl Oleate	17.49 ± 1.45	22.92 ± 0.82	*
C18:1	Methyl Vaccenate	3.26 ± 0.55	2.21 ± 0.13	*
C20:1	Methyl 11-eicosenoate	0.41 ± 0.09	0.27 ± 0.07	ns
C22:1	Methyl Erucate	0.16 ± 0.02	0.09 ± 0.04	ns
C24:1	Methyl Nervonate	0.11 ± 0.03	0.017 ± 0.003	*
**PUFA**				
C18:2	Methyl Linoleate	21.45 ± 1.39	31.02 ± 0.88	***
C18:3	Methyl Linolenate	0.19 ± 0.02	1.38 ± 0.06	***
C20:2	Methyl 11,14-eicosadienoate	0.62 ± 0.15	0.36 ± 0.11	*
C20:3	Methyl 8,11,14-eicosatrienoate	0.33 ± 0.04	0.73 ± 0.05	***
C20:4	Methyl Arachidonate	11.66 ± 1.00	5.93 ± 0.53	***
C22:4	Methyl Docosatetraenoate	0.64 ± 0.12	1.17 ± 0.03	*
C22:5	Methyl Docosapentaenoate	0.39 ± 0.11	0.76 ± 0.06	*
C22:6	Methyl Docosahexaenoate	1.53 ± 0.22	0.81 ± 0.08	*

Table 2. Fatty acids are grouped into saturated fatty acids (SFA), monounsaturated fatty acids (MUFA), and polyunsaturated fatty acids (PUFA). Data are reported as a percentage of the single fatty acid with respect to the total lipid content. Data are presented as mean ± SE, n = 6/group. The indicated *P* values were calculated by a T-Test and are reported as: *P ≤ 0.05, ***P ≤ 0.001, ns = not significant.

**Fig. 7:**
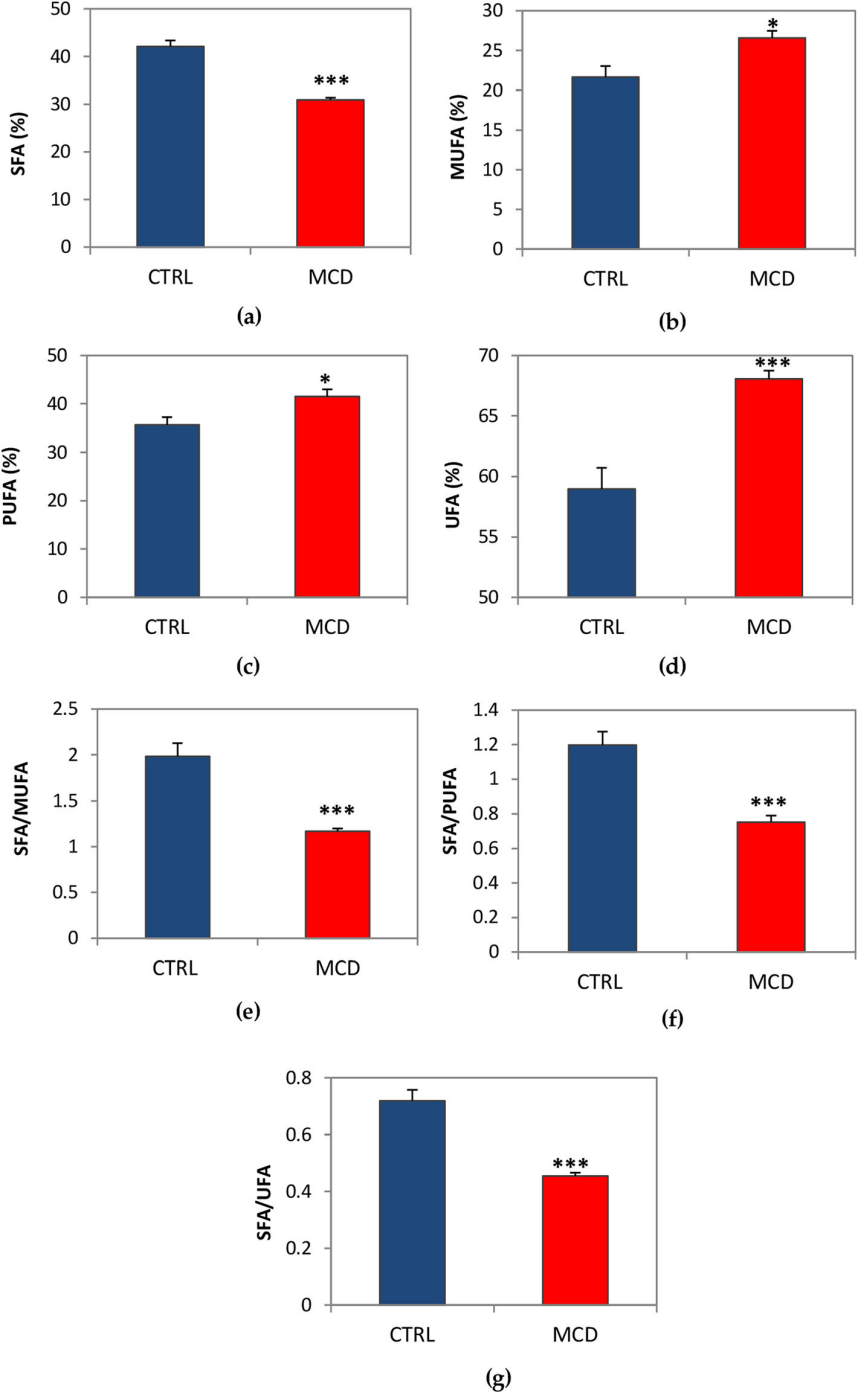
Percentages of SFA, MUFA, PUFA and UFA and ratio between the percentage of SFA and other types of fatty acids in livers from MCD rats. (**a**) SFA; (**b**) MUFA; (**c**) PUFA; (**d**) UFA; (**e**) SFA/MUFA ratio; (f) SFA/PUFA ratio; (g) SFA/UFA ratio. Data are presented as mean ± SE, *n* = 6/group. The *P* value was calculated by T-Test *P ≤ 0.05, ***P ≤ 0.001

The percentage of overall SFA was significantly reduced in MCD rats compared with the control group (Fig. 7A), while the MUFA, PUFA, and UFA (MUFA+PUFA) percentages were significantly increased in the MCD group with respect to the control rats (Fig. 7B, C and D). The ratio between SFA and the various types of unsaturated fatty acids was significantly lower in MCD rats (Fig. 7E, F and G).

The body weights of the MCD and control rats were evaluated before the sample collection to observe macroscopic differences in the two groups of animals. MCD rats showed significant reduced body weights when compared with the control rats. Aliquots of lipid extract were analyzed by Nile Red assay to quantify total lipid content. The measurement results were presented as total lipid/liver weight (mg/g). The Nile Red assay indicated a significant accumulation of fat in MCD-fed rats compared with the control rats. Liver injury was assessed in terms of the hepatic enzyme release in serum collected before liver excision and preservation. The ALT showed an increase in its value in MCD rats compared with the control rats. Moreover, higher levels of AST were measured in MCD rats with respect to the control rats. (Table 3).

**Table 3:** Body weights, hepatic total lipid content, and serum transaminase before liver procurement

	Control	MCD	*P
**Body weights (g)**	377.1 ± 25.97	259.2 ± 19.88	***
**Tissue total lipid (mg/g)**	0.05 ± 0.004	0.28 ± 0.05	***
**Serum ALT (mU/mL)**	31.67 ± 4.76	141.67 ± 15.12	***
**Serum AST (mU/mL)**	60.33 ± 5.06	120.5 ± 12.07	***

Table 3. Body weights, hepatic total lipid content, and serum transaminase before liver procurement: alanine aminotransferase (ALT) and aspartate aminotransaminase (AST). Data are presented as mean ± SE, *n* = 6/group. The indicated *P* values were calculated using a T-Test and are reported as: ***P ≤ 0.001.

## Discussion

It is well known that marginal livers are more susceptible to damage from ischemia and reperfusion, leading to early primary non-function, lower graft survival [22], and biliary complications [12, 23], and that ischemia/reperfusion injury tends to be more accentuated in livers submitted to prolonged cold preservation [24]. This study investigated the susceptibility of MCD livers to prolonged CS preservation. Previous studies by Ferrigno et al. have shown that the fatty acid profile affects cold ischemia injury. In fact, after 6 h of CS followed by 120 min of reperfusion, livers obtained from Obese Zucker rats exhibiting high SFA content displayed significantly more damage than livers from MCD rats, which instead had low SFA levels. Moreover, significantly better results were obtained in MCD rats concerning the bile flow, an index of good liver function, and the release of enzymes into the bile [11].

In the present study, cold storage was prolonged to 12 and 24 h; surprisingly, livers from MCD rats showing a six-fold higher content of fat with respect to the control livers survived optimally when subjected to cold preservation even up to 24 h. In fact, no differences in enzyme release (AST and LDH) both in the washout and in perfusate were detected in MCD livers after 6, 12, or 24 h of CS preservation. In this regard, Sun and colleagues [25] used male Wistar rats fed only with a choline deficient diet for 14, 28, or 42 days to simulate mild, moderate, or severe steatosis respectively, and they did not observe any significant differences in perfusate enzyme release, portal venous pressure (PVP), and biliary production in fatty livers with mild steatosis when compared with the control livers after both 6 or 12 h of CS preservation. In that case, the authors suggested that mild steatotic livers can be used for transplantation in a similar way that control livers can, whereas a moderate steatosis is more susceptible to the length of CS preservation, and thus livers with severe steatosis should be discarded [25]. In clinical practice, livers with mild steatosis (< 30%) are generally considered suitable for transplantation, livers with moderate steatosis (> 30%) may result in a less favorable outcome, and livers with severe steatosis (> 60%) are usually discarded [26]. However, Ferrigno et al. have shown that livers with mild steatosis from Obese Zucker rats did not survive after 6 h of CS preservation, while livers with moderate to severe steatosis from MCD rats showed better outcomes, demonstrating that the steatosis level is not the only determinant of transplant outcome and livers with moderate to severe steatosis, in certain conditions, possibly related to their lipidomic profile, could be suitable for transplantation [11].

No changes were detected in oxygen uptake during reperfusion in livers obtained from MCD rats when compared with control livers at any of the CS times considered. The same trend was also observed for the intra-hepatic PVP and the bile production during reperfusion time: there were no differences between MCD livers preserved at 6, 12, or 24 h of CS or between MCD and control livers. Most complications and bad outcomes after liver transplantation are related to portal hypertension [27], especially when marginal livers are employed. Similarly, suboptimal livers are more prone to post-transplant cholangiocyte damage and the development of ischemic-type biliary lesions. This condition is accompanied by the injury of the arterioles of the peribiliary vascular plexus, leading to cell apoptosis and necrosis of cholangiocytes after the cold ischemia and reperfusion procedure [28]. Moreover, regarding the bile flow, it has been demonstrated that in rats fed by a MCD diet, the protein expression of the hepatic transporter multidrug resistance-associated protein 2 (MRP2), localized at the apical membrane of hepatocytes, is surprisingly increased, thereby improving bile efflux in the bile ducts [29]. This finding can justify why in the present study no biliary damage was found in MCD rats when compared with control rats.

MCD livers have a significantly lower relative content of saturated fatty acids with respect to control livers, balanced by a significant increase in unsaturated fatty acids (MUFA, PUFA, and UFA) when compared with livers from Zucker rats, suggesting that the lipidomic profile may play a role in liver susceptibility to cold-ischemia injury [11]. Stanković and colleagues found similar results regarding the fatty acid composition of livers from mice fed by an MCD diet for 2 weeks. In fact, they observed a significant decrease in hepatic SFA content and PUFA arachidonic and docosahexaenoic acid, accompanied by an increase in oleic, docosapentaenoic, and linoleic acids [30, 31], as also observed in the present work. In particular, the present data on the SFA/PUFA, SFA/MUFA, and SFA/UFA ratios demonstrate that fatty acids containing one or more double bonds in their structure are very predominant in MCD livers, probably accounting for their ability to sustain CS preservation up to 24 h, as in the case of the control group. This hypothesis derives from the fact that fatty acids rich in double bonds show a lower tendency to crystallize than does SFA when exposed to low temperatures, thus creating less damage to hepatocytes during cold preservation [10]. In fact, the presence of a single double bond in the fatty acid chains is already sufficient to produce a great degree of membrane fluidization [32] since it introduces a kink, preventing close packing and thus enhancing fluidity [33] and allowing livers from MCD rats to withstand low temperatures. Moreover, the melting point of a fatty acid increases proportionally to its length, while the presence of double bonds in the fatty acid chain induces a decrease in its melting point, thereby avoiding the packaging of fatty acids in an ordered crystalline structure [32, 33]. It is fair to note that a high PUFA content due to diet may enhance cell susceptibility to H_2_O_2_ [34], accompanied by the alteration of membrane functionality and the activation of pro-inflammatory cells and pathways [35]. In this context, however, the importance of the membrane composition needs to be considered. A greater presence of saturated lipids surely contributes to stabilizing the structure, thereby causing less damage from oxidative stress. Nevertheless, low temperatures, such as those during CS preservation, are deleterious for saturated fatty acids, and livers from MCD rats, where the SFA relative content is reduced, are probably protected from this kind of damage. On the other hand, SFA are also involved in inflammatory processes. It has been demonstrated that overexposure to SFA in hepatic cells promotes pro-inflammatory cytokines expression and apoptosis initiation caused by endoplasmic reticulum (ER) impairment and oxidative stress [36, 37]. Similar data were achieved in vivo by Wang et al.: male Wistar rats fed by a diet rich in saturated fatty acids, but poor in unsaturated fatty acids, exhibited hepatic ER stress and liver damage [38]. In addition, studies by Joshi-Barve et al. have instead demonstrated that by treating hepatocytes with the saturated fatty acid, palmitic acid, the secretion of pro-inflammatory cytokine IL-8 is induced probably via the activation of the toll-like receptor 4 (TLR4) [39]. Fatty livers from MCD rats, here taken into account, are poor in SFA like palmitic acid, and this occurrence probably protects them from these kinds of damages. Moreover, the SFA behenic acid was significantly reduced in MCD rats when compared with the control rats. In this regard, studies conducted by Hein and co-workers showed that behenic acid can affect mitochondrial membrane potential, calcium homeostasis, as well as inducing cell death in oligodendrocytes [40]. This evidence could further explain why livers from MCD rats, which are poor in behenic acid, are less susceptible to ischemic damage.

It has been proven that MUFA induces triglyceride formation, but not apoptosis [41]. Studies by Nolan and Larter have also demonstrated that MUFA protects against SFA, mediating free fatty acid esterification into triglycerides or inducing detoxification by fatty acid oxidation [42]. The results obtained in the present study consistently show a significant increase in the monounsaturated fatty acid, oleic acid, in MCD rats, when compared with control rats. Other works have shown the beneficial effects of oleic acids in protecting hepatocytes from SFA-mediated ER stress and apoptosis [37]. Moreover, oleic acid has been confirmed to be more steatogenic, though less apoptotigenic, than palmitic acid in hepatocytes cell cultures [43]. Recent findings have demonstrated that mice pretreated with oleic acid improved their tolerance to hepatic I/R injury [44]. The proposed mechanism is that oleic acid could be able to downregulate the AKT/mTOR phosphorylation pathway and the consequent p65 nuclear translocations needed to initiate the inflammatory response [44]. These findings help to justify the low susceptibility of MCD livers to prolonged cold preservation. The advances provided in this work by the estimation of specific fatty acids has shown a reduction in arachidonic, eicosadienoic, and docosahexaenoic acids and derivatives, which may have detrimental implications as mediators of inflammatory damage. In particular, the reduction of arachidonic acid in livers from MCD rats might suggest that these kinds of livers also have a lower content of its major metabolite, 20-hydroxyeicosatetraenoic acid (20-HETE), which is known to contribute to exacerbating I/R injury in rats, as it is a potent vasoconstrictor of cerebral micro-vessels [45, 46]. AA was also found to be toxic in HepG2 cells overexpressing cytochrome P450 (CYP2E1), suggesting that it triggers lipid peroxidation, which is responsible for this toxicity [47]. However, a decrease in the glutathione (GSH) content in rats has already been documented after 2 weeks of MCD diet administration [16], and an increase in lipid peroxidation in MCD livers is reported in the present study when compared with controls. Therefore, the data reported probably suggest that the positive outcome of MCD livers after prolonged CS could be independent of oxidative stress and strictly linked to the lipidomic profile of these organs, which are rich in PUFA but poor in SFA.

### Study strength and limitations

This study has demonstrated that fatty livers from MCD rats, when subjected to 12 and 24 h CS preservation time, show good resistance to cold-ischemic injury despite having a six-fold increase in fat content with respect to the control livers. Moreover, a deep analysis was undertaken of the lipidomic profile of MCD and control livers, considering 21 specific fatty acids. As observed in the literature, this is the first time that such a thorough analysis of fatty acids present in the liver of rats fed by an MCD diet for 2 weeks has been carried out. However, this study also has some limitations. The analysis of the lipidomic profile was only performed before the preservation of the livers and not after CS or reperfusion time. However, the aim of the present work was not to evaluate any differences in the lipidomic profile after preservation, but to evaluate if the lipidomic profile could impact cold preservation. Currently, only a few studies in the literature have explored this issue. Kirac and colleagues presented an analysis of four fatty acids after 60-min warm hepatic ischemia followed by 60-min reperfusion in rats fed by standard food [48], but no other data are available on the analysis of the lipidomic profile after cold ischemia and reperfusion.

## Conclusions

The data presented in this study show that steatotic livers from MCD rats displayed no increased susceptibility to long-term CS preservation when compared with control livers; the differences observed in the fatty acid composition may be involved in survival after prolonged cold preservation. In particular, the decrease in SFA content in livers from MCD rats could protect them from the crystalline structure formation at low temperatures. This work provides important insights into the selection of steatotic livers for transplantation because it helps in understanding that it is not sufficient to carry out a quantitative analysis of the fat content in choosing the most adequate organ. In fact, the variations of the single fatty acids should also be taken into account. The lipid composition of human liver is strongly affected by diet; for example, it has been demonstrated that diet-induced long chain fatty acid ω-6/ω-3 ratio changes play an important role in the development of liver pathologies [49]. Such data suggest that the hepatic lipid profile may also have an impact on the outcome of liver preservation and transplantation since an evaluation and analysis of this profile may make it possible to more accurately predict the most suitable preservation method for each individual steatotic liver before transplantation.

## Data Availability

The datasets used and/or analysed during the current study are available from the corresponding author on reasonable request.

## Abbreviations

AA: Arachidonic acid
ALT: Alanine aminotransferase
AST: Aspartate aminotransaminase
CS: Cold storage
CYP2E1: Cytochrome P450
DCD: Donors after circulatory death
GC/MS: Gas chromatography-mass spectrometry
GSH: Glutathione
H&E: Hematoxylin and eosin
HCV: Hepatitis C virus
I/R: Ischemia/reperfusion
LDH: Lactate dehydrogenase
MCD: Methionine and choline deficient
MRP2: Multidrug resistance-associated protein 2
ER: Endoplasmic reticulum
MUFA: Monounsaturated fatty acid
NAFLD: Nonalcoholic fatty liver disease
PBS: Phosphate buffered saline
PNF: Primary non function
PUFA: Polyunsaturated fatty acid
PVP: Portal venous pressure
SFA: Saturated fatty acid
TLR4: Toll-like receptor 4
UFA: Unsaturated fatty acid
UW: University of Wisconsin
WO: Washout
